# Single high-dose buprenorphine for opioid craving during withdrawal

**DOI:** 10.1186/s13063-018-3055-z

**Published:** 2018-12-10

**Authors:** Jamshid Ahmadi, Mina Sefidfard Jahromi, Dara Ghahremani, Edythe D. London

**Affiliations:** 1 Substance Abuse Research Center, Shiraz University of Medical Sciences, Shiraz, Iran; 2 Department of Psychiatry and Biobehavioral Sciences, University of California at Los Angeles, Los Angeles, California USA; 3 Department of Molecular and Medical Pharmacology, University of California at Los Angeles, Los Angeles, California USA; 4 Brain Research Institute, University of California at Los Angeles, Los Angeles, California USA

**Keywords:** Buprenorphine, Craving, Opioid dependence, Opioid withdrawal

## Abstract

**Background:**

Opioid use disorder is one of the most prevalent addiction problems worldwide. Buprenorphine is used as a medication to treat this disorder, but in countries where buprenorphine is unavailable in combination with naloxone, diversion can be a problem if the medication is given outside a hospital setting.

**Objective:**

The objective of this research is to evaluate the effect of a single, high dose of buprenorphine on craving in opioid-dependent patients over 5 days of abstinence from use of other opioids. The primary goal was to determine the safety and efficacy of buprenorphine during withdrawal in a hospital setting.

**Methods:**

Ninety men who used opium, heroin, or prescribed opioids and met DSM-5 criteria for opioid use disorder (severe form) were randomized to three groups (*n* = 30 per group) to receive a single, sublingual dose of buprenorphine (32, 64, or 96 mg). The study was conducted in an inpatient psychiatric ward, with appropriate precautions and monitoring of respiratory and cardiovascular measures. Buprenorphine was administered when the patients were in moderate opiate withdrawal, as indicated by the presence of four to five symptoms. A structured clinical interview was conducted, and urine toxicology testing was performed at baseline. Self-reports of craving were obtained at baseline and on each of the 5 days after buprenorphine administration.

**Findings:**

Craving decreased from baseline in each of the three groups (*p* < 0.0001), with a significant interaction between group and time (*p* < 0.038), indicating that groups with higher doses of buprenorphine had greater reduction.

**Conclusions:**

A single, high dose of buprenorphine can reduce craving during opioid withdrawal; additional studies with follow-up are warranted to evaluate safety.

## Background

Buprenorphine, a partial agonist at mu-opioid receptors and an antagonist at delta- and kappa-opioid receptors, has been evaluated for the management of opioid use disorder [1–13]. Regarded as safer than methadone [5–7], buprenorphine at a dose of 8 mg is as effective as 60 mg of methadone [8]. Buprenorphine is well absorbed after sublingual administration [4, 9, 10], and its partial agonist action at mu-opioid receptors contributes to a safer profile of buprenorphine over methadone, with minimal respiratory depressant effects [14–26].

The purpose of this study was to assess the effects of single, high-dose buprenorphine administration (32, 64, or 96 mg) on opiate craving during initial abstinence. Craving is an essential feature of substance use disorders, as evidenced by its recent addition to the diagnostic criteria for these disorders in the Diagnostic and Statistical Manual of Mental Disorders, Fifth Edition (DSM-5; American Psychiatric Association) [3, 17, 20, 21], and it persists after detoxification to promote relapse [3, 17, 21, 22]. Buprenorphine was administered in a hospital setting to reduce the possibility of diversion of the medication, which is much more likely if the formulation does not include naloxone, which is included in some formulations for this purpose (e.g., Suboxone®). Such combined formulations are not available in Iran.

Doses of buprenorphine higher than those that are commonly administered clinically (i.e., 16–24 mg) were used to increase the effective half-life of the medication (the plasma elimination half-life of buprenorphine is 36–72 h after sublingual use) and to enhance mu-opioid receptor occupancy. A single high dose was examined because repeated buprenorphine administration in outpatients increases the possibility of dependence, diversion, and abuse (this is also based on our clinical experiences in Iran) [3, 18, 21, 22, 27, 28]. Buprenorphine was administered rather than methadone due to the risk of overdose with a single, high dose of methadone [22–24]. Common practice in our center is for opioid-dependent patients to undergo withdrawal as inpatients under supervision, to leave the hospital after detoxification without medication-assisted treatment, and then to return for psychosocial follow-up. If a patient requires medication when evaluated at follow-up, appropriate management, such as buprenorphine maintenance treatment, is initiated.

## Methods and materials

### Participants and procedures

This study was approved and monitored by the Committee of Ethics of Shiraz University of Medical Sciences; it adhered to the Declaration of Helsinki Ethical Principles for Medical Research Involving Human Subjects. At screening, participants were interviewed and examined for eligibility by a board certified psychiatrist. We explained the goals of the study, and guaranteed confidentiality. All the patients gave written informed consent prior to entering the study. The participants were male inpatients at the main psychiatric ward, where only men were hospitalized. Prior to admission, they had been abusing opium, heroin, and illicit or prescribed opioids for at least 1 year. Patients who met initial eligibility requirements on screening were administered the Structured Clinical Interview for DSM-5, Clinical Version (SCID-I), by a board certified psychiatrist to determine if they met the criteria for opioid use disorder (listed in DSM-5). Daily opioid abuse for at least 1 year was a requirement. Patients were excluded if they had substance use disorders involving drugs other than opioids (excluding tobacco), organic mental disorders, major medical diseases (hepatic, renal, cardiovascular, pulmonary, gastrointestinal, or malignant diseases), or any type of psychosis. The study was a double-blind randomized trial. The first 90 eligible treatment-seeking patients who referred to our ward were randomly assigned to the three buprenorphine arms (*n* = 30 per group).

Buprenorphine tablets (a single dose) were administered sublingually, while the patient was in moderate opioid withdrawal from opioids, as indicated by the presence of four or five symptoms of opioid withdrawal [3]. The buprenorphine doses tested were 32 mg, which is the maximum dosage currently used clinically, and two other doses that were twice and three times as much, respectively. The interview, examination, and questioning were performed at the treatment hospital. To enhance confidentiality and validity of the information, data were obtained from the patient in the absence of accompanying family or acquaintances.

A visual analog scale (VAS) that has been used previously [16–19] was used to measure the opioid craving, with a range of 0–10 (0 = no craving and 10 = severe desire, craving, or temptation all the time). Patients responded to the statement: Rate your craving over the past day. Measurements of craving were taken each morning. The hospital system covered sublingual tablets. Patients did not receive any form of compensation. During the hospitalization course, they did not receive any other methods of coping with craving (e.g., group sessions focused on relaxation/mindfulness/distraction, etc.).

A placebo group was not included because of the high possibility of severe withdrawal without active pharmacological treatment. The pills had the same shape and color. They were given in 8-mg increments. Everyone received the same number of pills. Placebo pills were used so that the patients did not know what dose they were receiving. Tablets were administered based on the tolerance of the patient.

Out of 90 patients, each group (30 patients) received 32 mg, 64 mg, or 96 mg of buprenorphine. Over the next 5 days, craving and adverse effects were evaluated. The degree of opioid craving was calculated and assessed through patients’ reports. Urine drug toxicology was carried out using thin-layer chromatography (TLC) before administration of the single dose, twice a week and at the end of the 5-day trial. To ensure safety, adverse effects, vital signs, respiration, and gastrointestinal effects were monitored every hour for the first day, and then every 6 h. For the current study, withdrawal was done in the hospital because we administered “high doses” instead of standard doses. We advocate using a single dose on an inpatient basis and then discharging the patients drug-free (without medication assistance treatment) and with an appointment for close psychosocial follow-up [2, 18, 20]. In any follow-up, if a patient needs medication, we start appropriate treatment such as buprenorphine maintenance treatment.

### Data analysis

Statistical analyses included both inferential and descriptive statistical methods. Data analysis was conducted using SPSS version 21. A repeated-measures two-way analysis of variance (ANOVA) was used, with day and group as the two factors and Greenhouse-Geisser correction for violation of sphericity. Post hoc *t* tests of differences in means were performed, and chi-square testing was used to test for differences in frequencies among the groups. The threshold for statistical significance was *p* < 0.05, both tails.

## Results

**Fig. 1:**
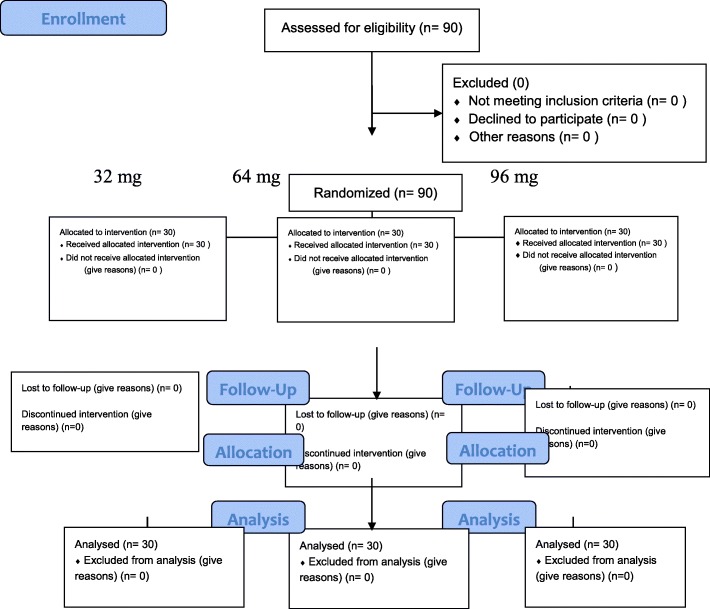
Consolidated Standards of Reporting Trials (CONSORT) flowchart of the patients in this trial

Data were collected from 90 men whose mean age was 32.85 ± 6.97 years. All the patients whom were screened entered the research study, and all of those who entered completed the trial (Fig. 1and Table 1). During the course of the study, no illicit opioid use was detected (based on daily interview and urine toxicology). All the patients had normal liver and kidney function before enrollment.

**Table 1:** CONSORT 2010 checklist of information to include when reporting a randomized trial

Section/topic	Item no.	Checklist item	Reported on page no.
Title and abstract			
	1a	Identification as a randomized trial in the title	1
	1b	Structured summary of trial design, methods, results, and conclusions (for specific guidance see CONSORT for abstracts)	1
Introduction			
Background and objectives	1a	Scientific background and explanation of rationale	2
	2b	Specific objectives or hypotheses	2
Methods			
Trial design	2a	Description of trial design (such as parallel, factorial) including allocation ratio	2
	2b	Important changes to methods after trial commencement (such as eligibility criteria), with reasons	2
Participants	2a	Eligibility criteria for participants	2
	2b	Settings and locations where the data were collected	2
Interventions	2	The interventions for each group with sufficient details to allow replication, including how and when they were actually administered	2
Outcomes	2a	Completely defined pre-specified primary and secondary outcome measures, including how and when they were assessed	2
	2b	Any changes to trial outcomes after the trial commenced, with reasons	2
Sample size	2a	How sample size was determined	2
	2b	When applicable, explanation of any interim analyses and stopping guidelines	NA
Randomization:			
Sequence generation	2a	Method used to generate the random allocation sequence	2
	2b	Type of randomization; details of any restriction (such as blocking and block size)	2
Allocation concealment mechanism	2	Mechanism used to implement the random allocation sequence (such as sequentially numbered containers), describing any steps taken to conceal the sequence until interventions were assigned	2
Implementation	2	Who generated the random allocation sequence, who enrolled participants, and who assigned participants to interventions	2
Blinding	2a	If done, who was blinded after assignment to interventions (for example, participants, care providers, those assessing outcomes) and how	2
	2b	If relevant, description of the similarity of interventions	NA
Statistical methods	2a	Statistical methods used to compare groups for primary and secondary outcomes	2
	2b	Methods for additional analyses, such as subgroup analyses and adjusted analyses	2
Results			
Participant flow (a diagram is strongly recommended)	3a	For each group, the numbers of participants who were randomly assigned, received intended treatment, and were analyzed for the primary outcome	2
	3b	For each group, losses and exclusions afterrandomization, together with reasons	3
Recruitment	3a	Dates defining the periods of recruitment and follow-up	3
	3b	Why the trial ended or was stopped	3
Baseline data	3	A table showing baseline demographic and clinical characteristics for each group	3
Numbers analyzed	3	For each group, number of participants (denominator) included in each analysis and whether the analysis was by original assigned groups	3
Outcomes and estimation	3a	For each primary and secondary outcome, results for each group, and the estimated effect size and its precision (such as 95% confidence interval)	3
	3b	For binary outcomes, presentation of both absolute and relative effect sizes is recommended	3
Ancillary analyses	3	Results of any other analyses performed, including subgroup analyses and adjusted analyses, distinguishing pre-specified from exploratory	3
Harms	3	All important harms or unintended effects in each group (for specific guidance see CONSORT for harms)	3
Discussion			
Limitations	6	Trial limitations, addressing sources of potential bias, imprecision, and, if relevant, multiplicity of analyses	3
Generalizability	6	Generalizability (external validity, applicability) of the trial findings	6
Interpretation	6	Interpretation consistent with results, balancing benefits and harms, and considering other relevant evidence	6
Other information			
Registration	6	Registration number and name of trial registry	6
Protocol	6	Where the full trial protocol can be accessed, if available	6
Funding	6	Sources of funding and other support (such as supply of drugs), role of funders	6

The Consolidated Standards of Reporting Trials (CONSORT) flow and the checklist for the study are shown in Fig. 1 and Table 1.

The three groups did not differ on demographic characteristics (Table 2). Table 3 presents craving scores of the three groups during the 5-day interval of treatment. A significant main effect of day (*F* (2, 2.16) = 199.96, *p* < 0.0001) but not group (*F* (2, 87) = 1.67, *p* = 0.194) and a significant group-by-day interaction (*F* (2, 4.32) = 2.52, *p* < 0.05) were found.

**Table 2:** Demographic characteristics of the patients

Group		32 mg *n* = 30 (33.33%)	64 mg *n* = 30 (33.33%)	96 mg *n* = 30 (33.33%)	Total *n* = 90 (100%)	Chi-square	*F*	df	*p* value^a^
Age (years)^b^ “M (SD).”		34.20 ± 7.30	32.83 ± 8.16	31.53 ± 5.035	32.85 ± 6.97		1.10	2	0.337
Drug abuse (years)^b^ “M (SD).”		9.75 ± 5.86	9.43 ± 6.52	10.50 ± 6.29	9.89 ± 6.17		0.232	2	0.794
Job^c^ “*n* (%)”	Unemployed	12 (40)	19 (63.3)	14 (40)	43 (47.8)	8.719		6	0.190
	Employed	2 (6.7)	3 (10.0)	2 (6.7)	7 (7.8)				
	Self-employed	16 (53.3)	7 (23.3)	16 (53.3)	39 (43.3)				
Education^c^ “*n* (%)”	Unable to read/write	1 (3.3)	1 (3.3)	0 (0)	2 (2.2)	4.918		8	0.766
	Primary school	11 (36.7)	8 (26.7)	10 (33.3)	29 (32.2)				
	High school	12 (40)	17 (56.7)	15 (50)	44 (48.9)				
	University education	6 (20)	4 (13.3)	4 (13.3)	14 (15.6)				
Marital status^c^ “*n* (%)”	Married	15 (50)	21 (70)	14 (46.7)	50 (55.6)	3.870		2	0.144
	Single	15 (50)	9 (30)	16 (53.3)	40 (44.4)				

^a^The three groups were compared by ANOVA (continuous measurement variables) and chi-square analysis (categorical data)

^b^Numbers tabulated indicate means ± standard deviation (SD)

^c^Numbers tabulated indicate how many participants were in each category

**Table 3:** Craving scores (means and standard deviations) of the three groups

Group (Buprenorphine, mg) Day	32 *n* = 30	64 *n* = 30	96 *n* = 30
Baseline	7.23 ± 3.51	6.93 ± 3.54	7.56 ± 3.53
Day 1	4.46 ± 3.95	4.96 ± 2.90	4.00 ± 2.75
Day 2	2.56 ± 3.23	3.03 ± 2.23	1.00 ± 1.74
Day 3	1.70 ± 2.39	0.900 ± 1.37	0.366 ± 0.927
Day 4	1.23 ± 1.86	0.300 ± 0.749	0.233 ± 0.727
Day 5	0.700 ± 1.14	0.100 ± 0.402	0.00 ± 0.00

Post hoc *t* tests revealed that the 32-mg group differed significantly from both the 64-mg and 96-mg groups, with lower craving observed for the higher dose groups. No significant differences were observed between the 64-mg and 96-mg groups, suggesting that the maximal effect on craving reduction was achieved with the 64-mg dose (Table 4).

**Table 4:** Post hoc *t* test *p* values of the three groups

Group	Baseline *p* value	Day 1 *p* value	Day 2 *p* value	Day 3 *p* value	Day 4 *p* value	Day 5 *p* value
32 vs 64	0.743	0.553	0.469	0.069	0.004	0.001
32 vs 96	0.716	0.579	0.017	0.003	0.002	0.000
64 vs 96	0.489	0.252	0.002	0.223	0.835	0.583

### Adverse effects

To ensure safety, side effects, vital signs, respiration, and gastrointestinal effects were measured and monitored every hour for the first day, and then every 6 h. Nine patients developed notable side effects. Two (both in the 96-mg group) developed significant hypotension (blood pressure of 75/50 and 80/45, respectively) and were treated with hydration. Two (both in the 32-mg group) developed nausea. Five (two in the 64-mg group and three in the 96-mg group) developed both nausea and vomiting. Patients who had nausea or vomiting were treated with antiemetic medications. No severe respiratory, cardiovascular, or gastrointestinal adverse effects were observed.

## Discussion

Buprenorphine has been evaluated extensively for the treatment of opioid use disorder [2–4]. In chronic use, it is considered for reducing craving and increasing long-term abstinence from illicit opioids [8].

Here we show that a single dose of buprenorphine (32 mg, 64 mg, and 96 mg) can provide a rapid, effective, and safe means of reducing opioid craving at 5 days post-treatment, 64 mg more so than 32 mg, with no greater effect at 96 mg. The comparable efficacy of the 64- and 96-mg dosages may reflect occupancy of mu-opioid receptors to the same degree over the short (5 days) post-treatment evaluation time. Doses higher than 16–24 mg are thought to increase the effective half-life of buprenorphine; therefore, high doses (64 mg and 96 mg) would be expected to be more effective than 32 mg, as observed here.

Administration of buprenorphine as a single large dose decreases concerns about compliance as well as the probability of dependence, diversion, and abuse. Moreover, cost considerations are favorable, especially when considering administration to outpatients without hospitalization. A single-dose treatment also is suited to transition to antagonist treatment, which could probably be started at an earlier time than with a traditional detoxification schedule lasting many days or even weeks. Moreover, it could also provide a more suitable titration of agonist treatment, potentially with lower maintenance doses being required. In patients who are unsuitable for or decline medication-assisted treatment, it would allow more rapid referral to either an intensive outpatient or residential treatment program.

Strengths of this study included the randomized clinical trial design and a reasonable number of patients, carefully diagnosed using DSM-5 criteria and urine drug screening tests. However, the study had some limitations, including its recruitment of men only. It would be important to know if the results are generalizable to both sexes and to determine the duration of the effect of single-dose buprenorphine on opioid craving. Administration of a high dose of buprenorphine may be far more likely to result in respiratory or cardiovascular complications in older patients with underlying occult disorders, especially sleep apnea, than in younger patients.

## Conclusions

The single-dose buprenorphine treatment provided safe and rapid treatment of opioid craving. The outcomes support further investigations of the use of a single high dose of buprenorphine as a safe and effective protocol to early treatment of these patients. Moreover, the findings support further investigations of a single dose to decrease opioid craving over more extended time frames.

## Abbreviations

DSM-5: Diagnostic and Statistical Manual of Mental Disorders, Fifth Edition
RCT: Randomized clinical trial
VAS: Visual analog scale
